# Association of anaemia with long-term mortality among patients with hypertensive crisis in the emergency department

**DOI:** 10.1080/07853890.2022.2128209

**Published:** 2022-10-07

**Authors:** Wook-Dong Kim, Byung Sik Kim, Jeong-Hun Shin

**Affiliations:** Department of Internal Medicine, Division of Cardiology, Hanyang University College of Medicine, Hanyang University Guri Hospital, Guri, Republic of Korea

**Keywords:** Hypertensive crisis, anaemia, mortality

## Abstract

**Background:**

Anaemia is frequent in patients with cardiovascular disease and is significantly associated with poor prognosis. However, the prognostic significance of anaemia in hypertensive crisis remains unknown. We conducted this study to determine whether anaemia is a risk factor for all-cause mortality in patients with hypertensive crisis visiting the emergency department (ED).

**Methods:**

This retrospective study included patients who visited the ED between 2016 and 2019 for hypertensive crisis, which was defined as systolic blood pressure ≥180 mmHg or diastolic blood pressure ≥110 mmHg. A total of 5,512 patients whose serum haemoglobin levels were checked were included in this study and were classified into three groups according to their serum haemoglobin levels at admission to the ED: moderate/severe anaemia (haemoglobin <11 g/dL), mild anaemia (haemoglobin 11 to <13 g/dL in men and 11 to <12 g/dL in women), and non-anaemia (haemoglobin ≥13 g/dL in men and ≥12 g/dL in women).

**Results:**

Among 5,512 patients, 665 (12.1%) and 668 (12.1%) were classified into the moderate/severe anaemia and mild anaemia groups, respectively. The three-year all-cause mortality rates in the moderate/severe anaemia, mild anaemia, and non-anaemia groups were 46.0, 29.2, and 12.0%, respectively. After accounting for relevant covariates, patients with moderate/severe anaemia group (hazard ratio [HR], 2.15; 95% confidence interval [CI], 1.75–2.64) and mild anaemia group (HR, 1.32; 95% CI, 1.07–1.63) had a higher risk of 3-year all-cause mortality than the non-anaemia group.

**Conclusion:**

Anaemia is independently associated with 3-year all-cause mortality in patients with hypertensive crisis. A comprehensive therapeutic approach through more in-depth examination and close follow up are required for patients with hypertensive crisis with anaemia.

**KEY MESSAGES**
- Anaemia is independently associated with 3-year all-cause mortality in patients with hypertensive crisis.
- A comprehensive therapeutic approach through more in-depth examination and close follow up are required for patients with hypertensive crisis with anaemia.

## Introduction

Hypertension is a common disease observed in approximately 30–45% of the adult population and is the leading cause of premature death despite considerable advances in antihypertensive treatments [1,2]. Hypertensive crisis, which is an abrupt and marked elevation in blood pressure (BP) is observed in 1–2% of patients with hypertension [3]. Although the hypertensive crisis is a serious condition, it is relatively common in the emergency department (ED), accounting for about 4.6% of all ED visits in the United States [4]. Advances in antihypertensive therapy have improved the treatment of hypertensive crisis. However, recent studies have shown that estimated annual mortality rates remain high in these patients [5,6]. While previous studies show that hypertension-mediated organ damage (HMOD) is related to poor prognosis [7–10], there are limited data on risk factors related to the prognosis of hypertensive crisis.

Anaemia is a haematologic abnormality commonly observed in clinical practice and is a well-known risk factor for poor outcomes in patients with cardiovascular diseases as well as the general population [11–14]. However, the clinical significance of anaemia in hypertensive crisis has not been studied. The aim of this study was to evaluate the association between anaemia and all-cause mortality in patients with hypertensive crisis who visited the ED.

## Materials and methods

### Study participants

**Figure 1.**
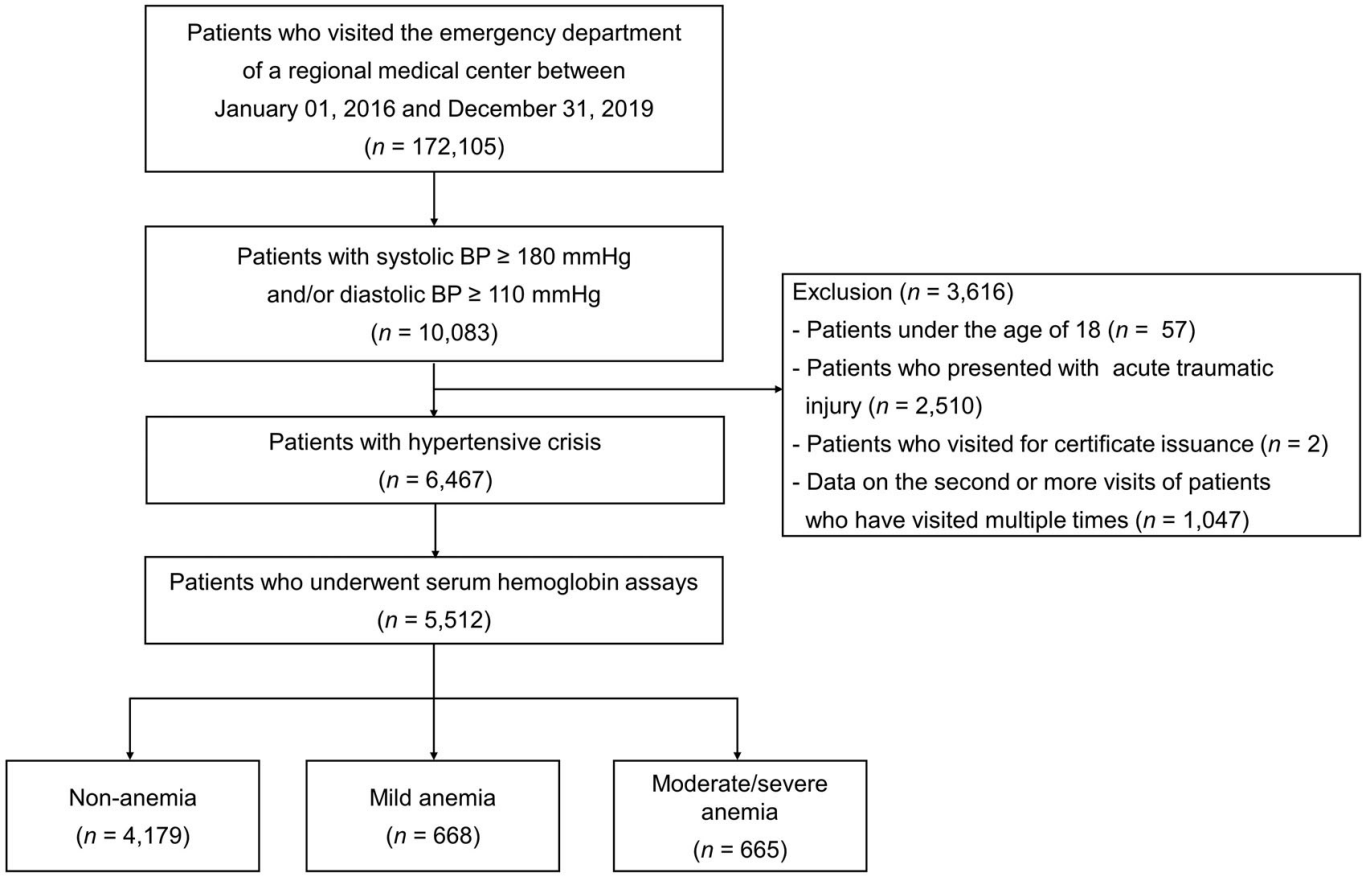
Flow chart illustrating patients with hypertensive crisis who were included in this study. BP: blood pressure

Among 172,105 patients who visited the ED of Hanyang University Guri Hospital, a regional emergency medical centre affiliated with the academic university hospital in Guri, Gyeonggi-do, Korea, between January 2016 and December 2019, 10,083 with systolic blood pressure (SBP) ≥180 mmHg or diastolic blood pressure (DBP) ≥110 mmHg were included in the study. Patients under the age of 18, those who came to the hospital due to acute trauma, or those who visited the ED for a medical certificate were excluded. If a patient visited the emergency room twice or more, only data from the first visit were included. Among 6,467 patients with hypertensive crisis, 5,512 patients who had undergone serum haemoglobin assays were included in the study (Figure 1).

### Data collection and outcomes

Data were collected from the hospital electronic medical records. A detailed description of study design and definition in this study was reported previously [6,9,10,15]. Demographic and clinical characteristics, presence of acute HMOD, and diagnostic test results at the index visit of ED were collected. In addition, admission, discharge, and death at the ED index visit, as well as ED revisits, readmission, and death during the follow-up periods were collected. Brachial BP was measured using an automated oscillometric BP device (Spot Vital Signs LXi, Welch Allyn, Skaneateles Falls, NY, USA). The event data of the included patients were obtained until the study end point (March 2021) or until death. The data on the occurrence and timing of the death of study participants were obtained from the National Health Insurance Service of South Korea. The study was performed in accordance with the Declaration of Helsinki and was approved by the Institutional Review Board of Hanyang University Guri Hospital.

### Definition of anaemia

Haemoglobin levels were measured using an automated haematology analyser (DXH 800, Beckman coulter, CA, USA). We classified the presence and severity of anaemia according to the World Health Organisation definition. Men with a haemoglobin level >13 g/dL and women with a haemoglobin level >12 g/dL were classified into the non-anaemia group. Mild anaemia is defined as a haemoglobin level from 11 to <13 g/dL for men and 11 to <12 g/dL for women; therefore, haemoglobin levels <11 g/dL in both men and women were classified as moderate/severe anaemia [16].

### Statistical analysis

All categorical data are presented as frequencies with percentages, while statistics for continuous variables are presented as means with standard deviations. The baseline characteristics were compared using the one-way analysis of variance or Kruskal–Wallis test followed by Tukey’s *post hoc* test or Dunn’s multiple comparison for continuous variables. In addition, the Chi-squared or Fisher’s exact test were used to examine categorical variables. The incidence was estimated as the total number of outcomes during the follow-up period divided by 1,000 person-years. Kaplan–Meier survival analyses and log-rank tests were used to compare the cumulative survival probability according to haemoglobin categories. The association between haemoglobin and 3-year all-cause mortality was determined using a Cox proportional hazards regression model with consideration of other clinically relevant variables. In addition to univariable analysis, three adjusted models were used. In Model 1, age and sex were adjusted as possible confounding variables. Model 2 included factors used in Model 1 plus BP, and medical histories of comorbidities (hypertension, diabetes mellitus, ischaemic stroke, haemorrhagic stroke, coronary artery disease, chronic kidney disease [CKD], and end-stage renal disease [ESRD]). Model 3 included factors used in Model 2 plus factors associated with subclinical HMOD (serum creatinine, proteinuria, cardiomegaly on chest radiography, and left ventricular hypertrophy [LVH] on electrocardiography [ECG]). Hazard ratios (HR) and 95% confidence intervals (CIs) were calculated for each Cox proportional hazards regression model. We performed additional subgroup analysis using multivariate Cox proportional hazards regression models for 6 binary specifications: age (≥65 or <65 years), sex, hypertensive emergency or hypertensive urgency, and presence or absence of a medical history of CKD or ESRD. We also conducted a restricted cubic spline curve analysis to demonstrate the continuous adjusted association between haemoglobin and all-cause mortality. A *p* < .05 was considered as statistically significant. All analyses were performed using the Statistical Package for the Social Sciences (SPSS) software (IBM SPSS Statistics for Windows, version 26; IBM Corp., Armonk, NY, USA).

## Results

### Baseline characteristics

A total of 5,512 patients were included in this study, and the follow-up data for up to 5.2 years were analysed. The median follow-up period was 2.9 years (interquartile range, 1.8–4.0 years). Among the enrolled patients, 665 (12.1%) had moderate/severe anaemia and 668 (12.1%) had mild anaemia. Table 1 displays the baseline characteristics according to the haemoglobin levels. The mean age was greater in patients with moderate/severe and mild anaemia than those in the non-anaemia group (71.6 ± 14.4 vs. 70.6 ± 14.4 vs. 59.7 ± 16.2, *p* < .001). Patients with mild anaemia had the lowest proportion of women (61.1% vs. 46.0% vs. 47.4%, *p* < .001). Additionally, patients with moderate/severe anaemia and mild anaemia had more comorbidities, including hypertension, diabetes mellitus, ischaemic stroke, coronary artery disease, heart failure, CKD, and ESRD as compared to those without anaemia. In the ED, SBP was higher in patients with moderate/severe anaemia and mild anaemia than in patients without anaemia (195 ± 22.5 vs. 193 ± 21.5 vs. 190 ± 22.1, *p* < .001), whereas DBP was higher in patients without anaemia than those in other groups (99.9 ± 19.1 vs. 103 ± 18.8 vs. 110 ± 16.4, *p* < .001). Patients with moderate/severe anaemia and mild anaemia also had worse laboratory findings in ED, including serum creatinine, estimated glomerular filtration rate, and troponin-I levels, than those without anaemia. In addition, dipstick proteinuria and cardiomegaly on chest radiography were more frequent in patients with moderate/severe anaemia and mild anaemia than in those without anaemia. Moreover, acute HMOD was predominantly observed in patients with moderate/severe anaemia (47.1% vs. 37.7% vs. 32.8%, *p* <.001).

**Table 1.** Baseline characteristics according to severity of anaemia.

	All patients (*n* = 5,512)	Moderate/Severe anemia^a^ (*n* = 665)	Mild anemia^b^ (*n* = 668)	Non-anemia^c^ (*n* = 4,179)	*p*-Value
Age, mean (SD)	62.5 (16.5)	71.6 (14.4)*	70.6 (14.4)↑	59.7 (16.2)	<.001
Women, *n* (%)	2,694 (48.9)	406 (61.1)	307 (46.0)	1,981 (47.4)	<.001
Medical history, *n* (%)					
Hypertension	2,953 (54.9)	492 (75.0)	469 (71.3)	1,992 (49.0)	<.001
Diabetes mellitus	1,473 (27.6)	341 (52.6)	269 (41.1)	863 (21.4)	<.001
Dyslipidemia	531 (10.0)	57 (8.9)	62 (9.6)	412 (10.3)	.498
Ischaemic stroke	441 (8.3)	96 (14.9)	76 (11.7)	269 (6.7)	<.001
Haemorrhagic stroke	156 (3.0)	20 (3.1)	22 (3.4)	114 (2.9)	.727
Coronary artery disease	508 (9.6)	95 (14.7)	89 (13.7)	324 (8.1)	<.001
Heart failure	231 (4.4)	78 (12.1)	52 (8.0)	101 (2.5)	<.001
Chronic kidney disease	459 (8.7)	254 (39.4)	125 (19.2)	80 (2.0)	<.001
End-stage renal disease	223 (4.2)	131 (20.4)	68 (10.5)	24 (0.6)	<.001
Social history, *n* (%)					
Cigarette smoking	956 (25.7)	79 (15.0)	87 (17.5)	790 (29.3)	<.001
Alcohol consumption	1,334 (35.3)	84 (15.9)	112 (22.5)	1,138 (41.4)	<.001
Triage vitals, mean (SD)					
SBP, mmHg	191 (22.2)	195 (22.5)*	193 (21.5)↑	190 (22.1)	<.001
DBP, mmHg	108 (17.4)	99.9 (19.1)†*	103 (18.8)↑	110 (16.4)	<.001
Laboratory tests					
Mean serum creatinine, mg/dL (SD)^d^	1.04 (1.13)	2.12 (2.56)†*	1.28 (1.42)↑	0.87 (0.55)	<.001
Mean eGFR, mL/min/1.73 m^2^ (SD)^d^	82.3 (27.5)	52.5 (34.4)†*	68.9 (28.7)↑	88.4 (22.4)	<.001
Troponin-I, ng/mL (SD)	0.17 (2.39)	0.22 (2.45)	0.44 (4.49)↑	0.12 (1.76)	.019
Hb, g/dL (SD)	13.5 (2.2)	9.43 (1.3)†*	11.8 (0.5)↑	14.4 (1.4)	<.001
Urinary analysis done, *n* (%)	3,611 (65.5)	466 (70.1)	449 (67.2)	2,696 (64.5)	.012
Proteinuria^e^, *n* (%)	1,185 (32.8)	283 (60.9)	195 (43.2)	707 (26.2)	<.001
Chest X-ray done, *n* (%)	5,154 (93.5)	634 (95.3)	626 (93.7)	3,894 (93.2)	.108
Cardiomegaly^f^, *n* (%)	719 (13.9)	142 (22.4)	108 (17.2)	469 (12.0)	<.001
ECG done, *n* (%)	4,844 (87.9)	633 (95.2)	611 (91.5)	3,600 (86.1)	<.001
LVH^g^, *n* (%)	595 (12.3)	90 (14.2)	82 (13.5)	423 (11.8)	.145
Acute HMOD, *n* (%)	1,935 (35.1)	313 (47.1)	252 (37.7)	1,370 (32.8)	<.001

Data are presented as *n* (%) or mean (SD), as appropriate. SD: standard deviation; SBP: systolic blood pressure; DBP: diastolic blood pressure; eGFR: estimated glomerular filtration rate; Hb: haemoglobin; ECG: electrocardiography; LVH: left ventricular hypertrophy; HMOD: hypertension-mediated organ damage.

^a^Moderate/severe anaemia is defined as a haemoglobin level <11 g/dL for men and women.

^b^Mild anaemia is defined as a haemoglobin level ranging from 11 to <13 g/dL for men and 11 to <12 g/dL for women.

^c^Non-anaemia is defined as a haemoglobin level ≥13 g/dL for men and ≥12 g/dL for women.

^d^These values did not include patients with end-stage renal disease.

^e^Proteinuria was defined as a dipstick urinalysis result ≥1+.

^f^Cardiomegaly on chest radiography was diagnosed when the ratio between the maximal horizontal cardiac diameter and maximal horizontal inner thoracic cage diameter was >0.5.

^g^LVH on ECG was diagnosed when it satisfied either the Cornell voltage criterion (the amplitude of R in aVL plus the amplitude of S or QS complex in V3 with a cut-off of >2.8 mV in men and >2.0 mV in women) or the Sokolow–Lyon criterion (the amplitude of S in V1 plus the amplitude of R in V5 or V6 ≥ 3.5 mV).

†*Post hoc p*: Moderate/severe anaemia group versus mild anaemia group, statistically significant (*p* < .05).

*Post hoc p: Moderate/severe anaemia group versus non-anaemia group, statistically significant (*p* < .05).

↑*Post hoc p*: Mild anaemia group versus non-anaemia group, statistically significant (*p* < .05).

### Outcomes of the index visit and follow-up

**Figure 2.**
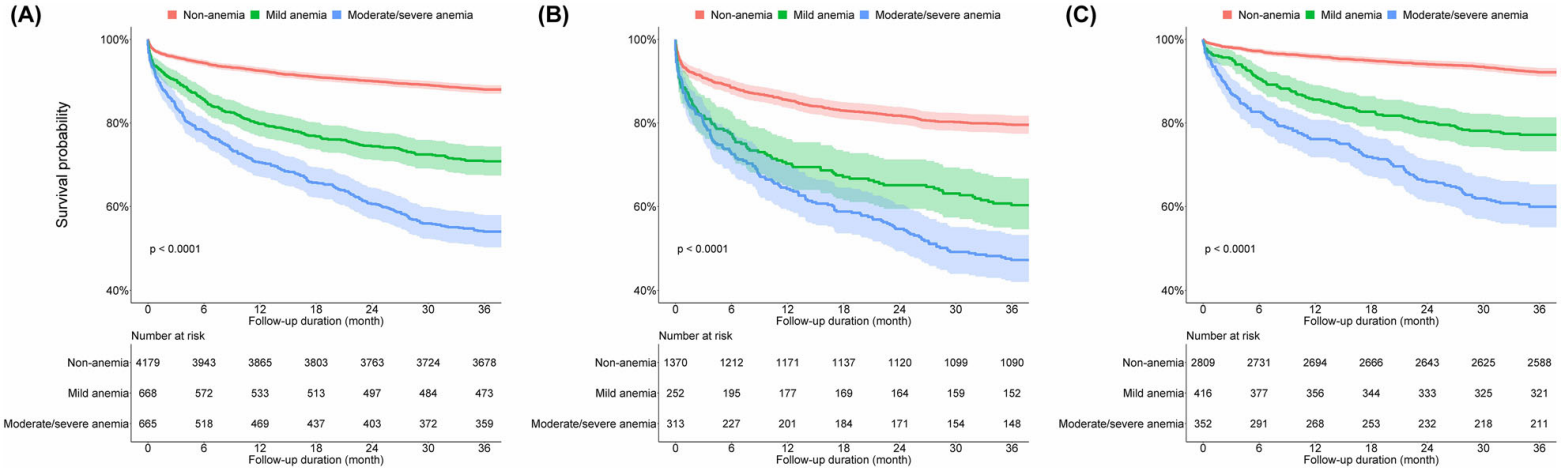
Kaplan–Meier curves comparing all-cause death free survival probability according to severity of anaemia. (A) All patients. (B) Patients with acute hypertension-mediated organ damage. (C) Patients without acute hypertension-mediated organ damage.

Overall, 2,914 (52.9%) patients were admitted, 2,050 (37.2%) were discharged, 542 (9.8%) were discharged against medical advice, and 6 (0.1%) died in the ED. Patients with moderate/severe anaemia and mild anaemia were more likely to be hospitalised than those in the non-anaemia group (70.5% vs. 56.9% vs. 49.4%, *p* < .001). At 3 years, the crude all-cause mortality rates were 46.0% for patients with moderate/severe anaemia, 29.2% for those with mild anaemia, and 12.0% for those without anaemia (Table 2). Similar findings were also observed in the incidence of all-cause mortality according to anaemia severity (Table S1). During the follow-up period, the cumulative death free survival rate was lowest in patients with moderate/severe anaemia and highest in patients without anaemia (Figure 2(A)). Similar trends were observed in patients with or without acute HMOD (Figure 2(B,C)). After adjusting for possible confounding variables such as age, sex, SBP, DBP, comorbidities, and components of subclinical HMOD, patients with moderate/severe anaemia (adjusted HR, 2.15; 95% CI, 1.75–2.64) and mild anaemia (adjusted HR, 1.32; 95% CI, 1.07–2.64) showed a significantly higher risk of 3-year all-cause mortality than those without anaemia (Table 3). A Cox proportional regression with restrictive cubic spline curve analysis showed a continuous association between haemoglobin levels and the risk of 3-year all-cause mortality (Figure S1). In the range of anaemia, the risk of 3-year all-cause mortality increased as the haemoglobin level decreased. The nadir for 3-year all-cause mortality risk was identified at a haemoglobin level of 15.0 g/dL.

**Table 2.** Outcomes of the index visit to the emergency department and mortality during the follow-up period according to severity of anaemia.

	All patients (*n* = 5,512)	Moderate/Severe anemia^a^ (*n* = 665)	Mild anemia^b^ (*n* = 668)	Non-anemia^c^ (*n* = 4,179)	*p*-Value
Outcomes of the index visit to the ED, *n* (%)					
Admission	2,914 (52.9)	469 (70.5)	380 (56.9)	2,065 (49.4)	<.001
Discharge	2,050 (37.2)	126 (18.9)	203 (30.4)	1,721 (41.2)	<.001
Discharge against medical advice	542 (9.8)	67 (10.1)	84 (12.6)	391 (9.4)	.034
Death in the emergency department	6 (0.1)	3 (0.5)	1 (0.2)	2 (0.0)	.013
Mortality, *n* (%)					
1-Month mortality	229 (4.2)	62 (9.3)	44 (6.6)	123 (2.9)	<.001
3-Months mortality	351 (6.4)	107 (16.1)	65 (9.7)	179 (4.3)	<.001
1-Year mortality	645 (11.7)	196 (29.5)	135 (20.2)	314 (7.5)	<.001
3-Year mortality	1,002 (18.2)	306 (46.0)	195 (29.2)	501 (12.0)	<.001

Data are presented as *n* (%). ED: emergency department.

^a^Moderate/severe anaemia is defined as a haemoglobin level <11 g/dL for men and women.

^b^Mild anaemia is defined as a haemoglobin level ranging from 11 to <13 g/dL for men and 11 to <12 g/dL for women.

^c^Non-anaemia is defined as a haemoglobin level ≥13 g/dL for men and ≥12 g/dL for women.

**Table 3.** The hazard ratios for 3-year all-cause mortality according to severity of anaemia among patients with hypertensive crisis.

	Unadjusted HR (95% CI)	Model 1^a^ (95% CI)	Model 2^b^ (95% CI)	Model 3^c^ (95% CI)
Non-anemia^d^	REF	REF	REF	REF
Mild anemia^e^	2.70 (2.29–3.18)	1.48 (1.25–1.76)	1.44 (1.20–1.72)	1.32 (1.07–1.64)
Moderate/severe anemia^f^	4.75 (4.12–5.48)	2.80 (2.42–3.25)	2.49 (2.10–2.96)	2.13 (1.73–2.63)

HR: hazard ratio; CI: confidence interval; REF: reference.

^a^Model 1: Adjustment for age and sex.

^b^Model 2: Adjustment for age, sex, systolic blood pressure, diastolic blood pressure, and comorbidities (hypertension, diabetes mellitus, ischaemic stroke, haemorrhagic stroke, coronary artery disease, chronic kidney disease, and end stage renal disease).

^c^Model 3: Adjustment for age, sex, systolic blood pressure, diastolic blood pressure, and comorbidities (hypertension, diabetes mellitus, ischaemic stroke, haemorrhagic stroke, coronary artery disease, chronic kidney disease, and end stage renal disease) and components of subclinical hypertension-mediated organ damage (serum creatinine, proteinuria, cardiomegaly on chest radiography, and left ventricular hypertrophy on electrocardiography).

^d^Non-anaemia is defined as a haemoglobin level ≥13 g/dL for men and ≥12 g/dL for women.

^e^Mild anaemia is defined as a haemoglobin level ranging from 11 to <13 g/dL for men and 11 to <12 g/dL for women.

^f^Moderate/severe anaemia is defined as a haemoglobin level <11 g/dL for men and women.

**Figure 3.**
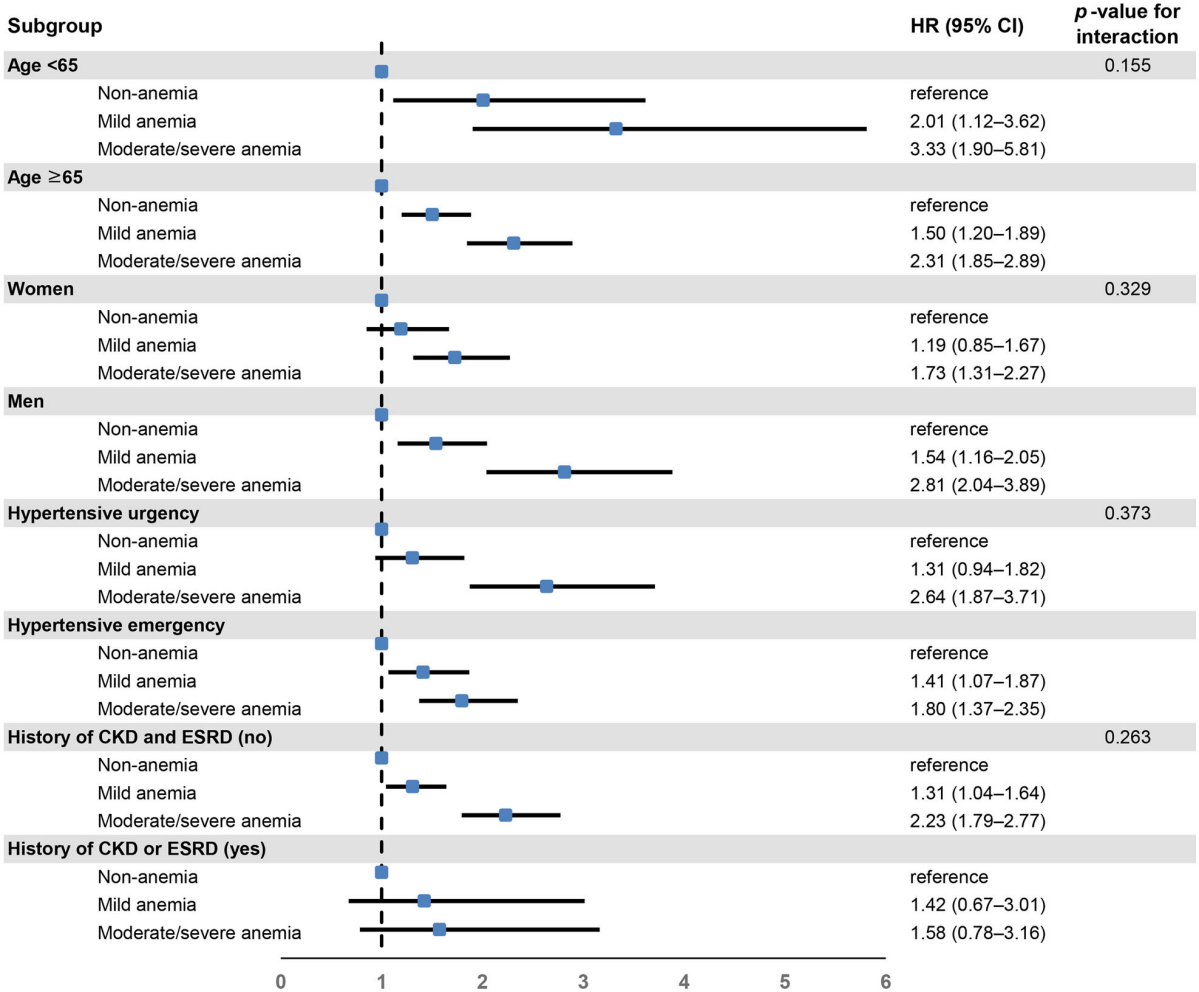
Subgroup analysis for risk of 3-year all-cause mortality according to severity of anaemia. Hazard ratios were adjusted for age, sex, systolic blood pressure, diastolic blood pressure, comorbidities (hypertension, diabetes mellitus, ischaemic stroke, haemorrhagic stroke, coronary artery disease, chronic kidney disease, and end-stage renal disease), and components of subclinical hypertension-mediated organ damage (serum creatinine, proteinuria, cardiomegaly on chest radiography, and left ventricular hypertrophy on electrocardiography). CKD: chronic kidney disease; ESRD: end-stage renal disease; HR: hazard ratio; CI: confidence interval.

In addition, we performed a subgroup analysis stratified by age (<65 or ≥65 years), sex (women or men), hypertensive urgency or hypertensive emergency, and the presence or absence of a medical history of CKD or ESRD. The HR and 95% CI for all-cause mortality according to haemoglobin categories (moderate/severe anaemia, mild anaemia, and non-anaemia) were similar in all subgroups, except for women, patients with hypertensive urgency, and patients with CKD or ESRD. In the subgroup of women and of patients with hypertensive urgency, the risk of 3-year all-cause mortality was not significantly higher in patients with mild anaemia than in those without anaemia; however, the risk of mortality was significantly higher in patients with moderate/severe anaemia than in those without anaemia. In patients with CKD or ESRD, the risk of 3-year all-cause mortality in patients with moderate/severe and mild anaemia was not significantly higher than that in those without anaemia (Figure 3).

## Discussion

In this study, anaemia was associated with a higher risk of 3-year all-cause mortality independent of other clinically relevant variables in patients who presented with the hypertensive crisis in the ED. The risk of 3-year all-cause mortality increased with the severity of anaemia. This prognostic significance was consistently observed regardless of age (<65 or ≥65 years) and the presence of acute HMOD. Our study suggests that even mild anaemia had a prognostic impact on mortality in patients with hypertensive crisis, special attention, more comprehensive therapeutic approach through in-depth examination and close follow-up on this high-risk group is required.

Anaemia has been reported as a risk factor for all-cause mortality not only in the general population but also in patients with cardiovascular disease, including heart failure, acute coronary syndrome, and peripheral artery disease [14,17–19]. Moreover, anaemia is associated with LVH, the severity of coronary artery disease, and progression of chronic kidney disease [20–22]. In this study, we demonstrated that mild anaemia showed a HR of 1.32 (95% CI, 1.07–2.64) and moderate/severe anaemia showed an HR of 2.15 (95% CI, 1.75–2.64) for all-cause mortality using a fully adjusted model: these increased risks were consistent in most subgroups. This may be evidence that highlights the importance of evaluating the excess risk of death in patients with hypertensive crisis with anaemia.

Several possible explanations regarding association between anaemia and all-cause mortality in patients with hypertensive crisis were observed in this study. First, patients with moderate/severe and mild anaemia were older and had acute HMOD more frequently. Second, the more severe the anaemia, the higher the number of comorbidities including hypertension, diabetes, ischaemic stroke, coronary artery disease, heart failure, CKD, and ESRD. Third, patients with more severe anaemia presented with worse diagnostic test results, which was associated with subclinical HMOD such as elevated creatinine levels, proteinuria, cardiomegaly on chest radiography, and LVH on ECG. However, the association between anaemia and increased risk of all-cause mortality remained, even after adjusting for the aforementioned confounding variables. Another explanation is that tissue hypoxia in patients with anaemia triggers an increased myocardial workload, leading to adverse outcomes [23]. Further studies are needed to clarify the associations and to understand the underlying mechanisms between anaemia and mortality in patients with hypertensive crisis.

One of the interesting results of this study was that anaemia and all-cause mortality were not statistically significant in the subgroup analysis of patients with CKD. These results were also reported in previous papers that studied the relationship between anaemia and mortality or the relationship between anaemia and cardiovascular outcomes in patients with CKD [24,25]. Maintaining high haemoglobin levels in CKD patients may increase the risk of hypertension and stroke, which may increase mortality [26,27]; this may explain the lack of statistical significance between anaemia and all-cause mortality in subgroups of CKD patients. However, this result must be interpreted cautiously because the insufficient number of patients precluded the detection of statistical significance in the CKD subgroup. In addition, as this study did not investigate the cause of death other than all-cause mortality or the occurrence of cardiovascular disease, additional evaluation is needed in future studies to explain these results.

This study had several limitations. First, since this was a retrospective observational study, the results cannot explain the causal relationship between anaemia and all-cause mortality. Second, although the study was based on the reliable registry and electronic medical chart data, compared with the accuracy and completeness of data examined in prospective studies, those of retrospective data are insufficient. Among patients with hypertensive crisis, only those patients who underwent blood tests including haemoglobin assays were included in the study; therefore, the results of this study are not representative of all patients with hypertensive crisis and the possibility of selection bias cannot be excluded. Moreover, we used only baseline haemoglobin levels and did not consider follow-up haemoglobin results or subsequent interventions such as treatment for anaemia or hypertension as well as comorbid conditions or physical function status. Third, anaemia can have several causes such as iron deficiency, vitamin B12/Folate deficiency, chronic inflammation, and bone marrow disease. However, in this study, we did not investigate the causes of anaemia or perform additional investigations to differentiate the causes of anaemia such as serum iron, ferritin, vitamin B12/Folate levels, and inflammatory markers such as C-reactive protein or erythrocyte sedimentation rate. Finally, we could not identify cardiovascular and renal events or cardiovascular and cancer mortality because the National Health Insurance Service data did not provide information on the causes of death. However, data regarding all-cause mortality and date of death were mathematical because they were obtained from the National Health Insurance Service, which covers the entire Korean population. Further prospective studies are needed to identify the appropriate diagnostic and therapeutic strategies related to adverse cardiovascular events or deaths according to anaemia in patients with hypertensive crisis. Despite these limitations, our study has several strengths. First, this is the first large-scale study to reveal the association between anaemia and long-term mortality in patients with hypertensive crisis. Second, even in the mild anaemia group, a statistically significant difference could be confirmed when compared with the patients without anaemia. Many studies have been conducted on the clinical significance of severe anaemia (haemoglobin 9–10 g/dL or less); however, there have been few studies on mild anaemia. Our study found that mild anaemia was also associated with long-term mortality in patients with hypertensive crisis.

In conclusion, the risk of long-term mortality was higher in patients with moderate/severe and mild anaemia. Special attention, more comprehensive therapeutic approach through an in-depth examination and close follow-up are required for patients with hypertensive crisis with anaemia.

## Data Availability

The datasets generated during and/or analysed during the current study are available from the corresponding author upon reasonable request.
